# Is the ratio of pleural mesothelioma mortality to pleural cancer mortality approximately unity for Italy? Considerations from the oldest regional mesothelioma register in Italy

**DOI:** 10.1038/sj.bjc.6600363

**Published:** 2002-06-17

**Authors:** G Gorini, E Merler, E Chellini, E Crocetti, A S Costantini

**Affiliations:** Unit of Occupational and Environmental Epidemiology, Center for Study and Prevention of Cancer (CSPO), Florence, Italy; Unit of Clinical and Descriptive Epidemiology, Center for Study and Prevention of Cancer (CSPO), Florence, Italy; Occupational Health Unit, Local Health Authority of Padua, Italy

## Abstract

doi:10.1038/sj.bjc.6600363 www.bjcancer.com

© (2002) Cancer Research UK

## Sir

In the article published in 1999 in the *British Journal of Cancer*, Peto *et al* (1999) estimated mesothelioma mortality projections in Western Europe for the period 1995–2029. The estimates were based on the ratio of pleural mesothelioma mortality to pleural cancer mortality (ICD 163) in males. In Britain it has been 1.6 : 1, for other Western European countries the ratio was assumed to be 1 : 1. In fact in these countries pleural cancer death rates are the only routinely available national data that correspond closely to mesothelioma incidence (Peto, 1999; Merler *et al*, 1999).

We calculated the ratio of mesothelioma to pleural cancer mortality in males in the period 1994–1999 for Tuscany, an Italian region of about 3.5 million inhabitants, using data from the Tuscan mesothelioma register and the Tuscan mortality register.

The Tuscan mesothelioma register records pleural malignant mesothelioma cases of Tuscan residents by means of histological, cytological, or clinical (radiography or computed tomography) diagnosis. The Tuscan mesothelioma register began its activity in 1988 and has provided consistent and reliable incidence data since 1994.

The Tuscan mortality register records and codes death certificates of Tuscan residents since 1987.

In the period 1994–1999, 228 male pleural mesothelioma cases were recorded in the Tuscan Mesothelioma Register. On 31 December, 1999, 188 cases had died, 40 were alive. In the same period, 256 male pleural cancer (ICD 163) deaths were recorded in the Tuscan Mortality Register.

Eighty-two per cent (154 out of 188) of male mesothelioma deaths recorded in Tuscan Mesothelioma Register were correctly coded as pleural cancer on the death certificate; 9.0% were coded as lung cancer, 2.1% as peritoneal or retroperitoneal cancer, 2.7% as other or unspecified cancers, 4.3% as non-malignant causes. In Tuscany the proportion of mesothelioma deaths correctly coded as pleural cancer (82%) was higher than that recorded in Britain (55%) (Peto, 1999), and similar to those estimated in France (75%) (Brochard *et al*, 1995), and in a sample of 523 Italian mesothelioma cases (75%) (Bruno *et al*, 1996).

Sixty per cent (154 out of 256) of all deaths coded ICD 163 were also recorded in the mesothelioma register. This proportion was 70% in France (Brochard *et al*, 1995), and 89% in Britain (Peto, 1999). It cannot be calculated accurately for all of Italy because of lack of reliable mortality and incidence data.

For males in the period 1994–1999 in Tuscany the ratio of pleural malignant mesothelioma mortality to pleural cancer mortality is therefore 0.73 : 1 (0.60 out of 0.82). The ratio was 1.62 : 1 (0.89 out of 0.55) in Britain (Peto, 1999) and 0.93 : 1 in France (0.70 out of 0.75) (Brochard *et al*, 1995).

In addition, we investigated whether the subgroup of pleural cancer deaths not recorded in the Tuscan Mesothelioma Register among residents in Florence and Prato provinces were recorded in the Tuscan Cancer Register, which collects cancer cases in these provinces. Among the 22 death certificates of residents in Prato or Florence provinces that were coded ICD 163 and not recorded in the Tuscan Mesothelioma Register, three cases were not recorded in the Tuscan Cancer Register, 11 cases were recorded as lung cancer, three as other cancers, and five cases (23%) as pleural cancer without histological diagnosis and without the word ‘mesothelioma’ in any medical report.

The low ratio of mesothelioma to pleural cancer mortality for Tuscany is above all due to the poor quality of death certificates coded ICD 163, as the linkage with the Tuscan Cancer Register shows. The accuracy of death certificates coded ICD 163 is not likely high for all of Italy. For this reason, Italian mesothelioma registers do not record mesothelioma cases based on death certificates only (Chellini *et al*, 1996).

In recent years, according to an Italian regulation (law 277/1991), a national mesothelioma register has been developed (Nesti *et al*, 2001). Its activity is based on case collection made at a regional level. The standardisation of the registration procedures is going on, and all of Italy is not yet covered by the register.

In the future years data from the national mesothelioma register will allow to calculate the ratio of mesothelioma mortality to pleural cancer mortality for a more extended area than Tuscany, and to make reliable projections of mesothelioma mortality in Italy.
